# Saltmarsh Boundary Modulates Dispersal of Mangrove Propagules: Implications for Mangrove Migration with Sea-Level Rise

**DOI:** 10.1371/journal.pone.0119128

**Published:** 2015-03-11

**Authors:** Jennifer M. Peterson, Susan S. Bell

**Affiliations:** Department of Integrative Biology, University of South Florida, Tampa, Florida, United States of America; Texas A&M University at Galveston, UNITED STATES

## Abstract

Few studies have empirically examined the suite of mechanisms that underlie the distributional shifts displayed by organisms in response to changing climatic condition. Mangrove forests are expected to move inland as sea-level rises, encroaching on saltmarsh plants inhabiting higher elevations. Mangrove propagules are transported by tidal waters and propagule dispersal is likely modified upon encountering the mangrove-saltmarsh ecotone, the implications of which are poorly known. Here, using an experimental approach, we record landward and seaward dispersal and subsequent establishment of mangrove propagules that encounter biotic boundaries composed of two types of saltmarsh taxa: succulents and grasses. Our findings revealed that propagules emplaced within saltmarsh vegetation immediately landward of the extant mangrove fringe boundary frequently dispersed in the seaward direction. However, propagules moved seaward less frequently and over shorter distances upon encountering boundaries composed of saltmarsh grasses versus succulents. We uniquely confirmed that the small subset of propagules dispersing landward displayed proportionately higher establishment success than those transported seaward. Although impacts of ecotones on plant dispersal have rarely been investigated *in situ*, our experimental results indicate that the interplay between tidal transport and physical attributes of saltmarsh vegetation influence boundary permeability to propagules, thereby directing the initial phase of shifting mangrove distributions. The incorporation of tidal inundation information and detailed data on landscape features, such as the structure of saltmarsh vegetation at mangrove boundaries, should improve the accuracy of models that are being developed to forecast mangrove distributional shifts in response to sea-level rise.

## Introduction

Impacts of climate change may be displayed by a variety of responses at the individual population, species, community, or ecosystem level. Populations are predicted to track their niche and shift predictably towards suitable areas [1, 2], but life-history traits and dispersal characteristics may limit a species’ ability to migrate as climatic variables change [3–5]. In certain cases, rare dispersal events (e.g., long-distance dispersal) may be essential for the persistence of some plant taxa [6]. Forecasting the ecological impacts or possible extinction of taxa due to climate change remains challenging given current limitations to modeling approaches, including quality of data [6] and the need to understand better the spatio-temporal dynamics of populations under future climate scenarios [3, 7]. Experimental data focused on life-history traits, dispersal and colonization can inform modeling efforts by contributing unique insight into the adaptive capacity of taxa when faced with changing climatic conditions.

Although directional dispersal of individuals towards those areas suitable for sustaining populations has often been discussed [4, 8] and modeled [9–11], few studies, especially ones targeting coastal plants, have empirically examined the suite of mechanisms that underlie such distributional shifts [12, 13]. Several different types of plant distributional shifts are expected to occur as global temperatures increase; latitudinal and altitudinal shifts of plant distributions have been frequently studied [14–16]. For example, latitudinal shifts in the distribution of coastal plant taxa, such as mangroves (dominant emergent vegetation found along sheltered subtropical/tropical coastlines) have been documented using historical maps and satellite imagery [17, 18]. Interestingly, coastal plants are also expected to migrate landward toward higher tidal elevations in response to sea-level rise induced by global warming [19, 20], and examples of such landward shifts have been documented during past sea-level rise events [21, 22]. Some coastal plant taxa are capable of vegetative expansion, i.e., grasses can spread via runners or stolons; however, the range expansion by other coastal plant taxa, including mangrove trees, depends upon the dispersal of seeds or propagules (i.e., dispersal units or diaspores). Landward shifts in the spatial distributions of mangroves have been recently documented across their global range [19, 23], in response to climate drivers [24]. An intriguing aspect of such landward migration is that it runs counter to the prevailing paradigm that mangrove propagules tend to disperse seaward [25, 26]. Thus, investigating the dispersal trajectory and establishment success of mangrove propagules at landward margins remains an essential task as the adaptive capacity of forests to shift toward higher elevations is being assessed [27, 28].

Assessing the capacity for plants to shift distributions that match climatic conditions requires, in part, quantitative information on both physical and biological factors that influence dispersal distances and propagule establishment. Dispersal characteristics of mangroves suggest possible pathways for mangrove recruitment into higher elevation areas, albeit thought to be infrequent [29, 30]. Mangrove propagules are hydrochorous [31], and effective dispersal is limited by water level [32, 33], propagule buoyancy and viability [31]. During occasional high water events, such as storm surges [11] and extreme high tides, tidal cover extends beyond the inland edge of fringing mangroves. Such tidal transport can potentially move propagules landward [34]. However, if transported tidally beyond the mangrove edge, propagules may encounter a vegetation boundary frequently occupied by saltmarsh forbs, shrubs, and grasses which vary in architectural structure [23, 29, 34] and permeability of the saltmarsh boundary (*sensu* boundary permeability [35]) to mangrove propagules may assume critical importance during such high water events.

The spatial arrangement of saltmarsh vegetation adjacent to mangrove forest edges provides an optimal setting for investigating the role of vegetation boundaries in regulating dispersal across landscapes. Principles of boundary function have been explored theoretically [35–37], and the responses of mobile organisms to boundary permeability have been the foci of studies [37, 38]. In contrast, few empirical assessments of boundary permeability to passively transported organisms exist [39]. Specifically, whether vegetation matrices act as an ecological filter, reducing plant dispersal, remains relatively unexplored [39, 40], although, this has been experimentally demonstrated for wind-dispersed seeds traversing forest boundaries [39]. If mangrove propagule dispersal is modulated by saltmarsh plant structure [34], then observed shifts in mangrove distribution under changing climatic conditions should reflect a response to a template established by marsh boundary permeability.

To evaluate the role of biotic boundaries in directing the landward migration of mangrove forests, we follow the fate of mangrove propagules at saltmarsh boundaries that differed in permeability and examine potential mechanisms operating on dispersing stages of mangroves that may influence spatial patterns of recruitment. We determine both direction and distance that propagules of the black mangrove, *Avicennia germinans*, move in tidal waters upon encountering a saltmarsh boundary at the leading edge of mangrove expansion. Using a field experiment, we compare dispersal patterns of mangrove propagules encountering different boundary conditions as represented by two types of structurally-distinct saltmarsh plants, i.e., succulent and grass. *A priori*, we expected a net seaward direction of mangrove propagule movement [25], regardless of the type of saltmarsh vegetation encountered by propagules and therefore quantifying landward dispersal was a primary objective. We also compare propagule retention among different types of saltmarsh taxa. Any increase in incidence of propagule sequestration by saltmarsh taxa should reduce the seaward transport of propagules away from the inland edge of the mangrove forest [26, 34]. If so, then the number of potential colonizers positioned at the leading edge of forest migration should be enhanced. Building upon previous observations, which demonstrated that structural complexity of a saltmarsh grass exceeded the complexity of succulent taxa and that structural complexity enhances mangrove propagule entrapment [34], we expected propagules obstructed by boundaries containing succulent vegetation to be dispersed seaward more readily than propagules encountering boundaries composed of saltmarsh grass.

Finally, saltmarsh boundary composition was predicted to alter the dispersal trajectories and subsequent deposition site of propagules along the tidal gradient, which, in turn, was expected to influence the probability of mangrove propagule stranding and seedling establishment [26]. Establishment success of *A*. *germinans* requires stranding which facilitates root initiation and enables propagules to anchor in sediments [31]. We therefore record the post-dispersal establishment of propagules. A greater proportion of propagules transported landward was expected to strand and initiate rooting relative to that transported seaward to lower tidal elevations, where increased inundation might reduce stranding and inhibit root development [31]. Combined, our investigations elucidate the effect of boundary species composition on spatial patterns of mangrove dispersal and the consequential impact of propagule deposition site on successful seedling establishment, two critical processes in mangrove migration in response to sea-level rise.

## Methods

### Ethics Statement

This field study was conducted within the Rookery Bay National Estuarine Research Reserve with their permission and in accordance with all research requirements of this entity. The Reserve is managed by the Florida Department of Environmental Protection’s Coastal Office in cooperation with the National Oceanic and Atmospheric Administration (NOAA). No protected species were sampled as part of our investigation.

**Fig 1:**
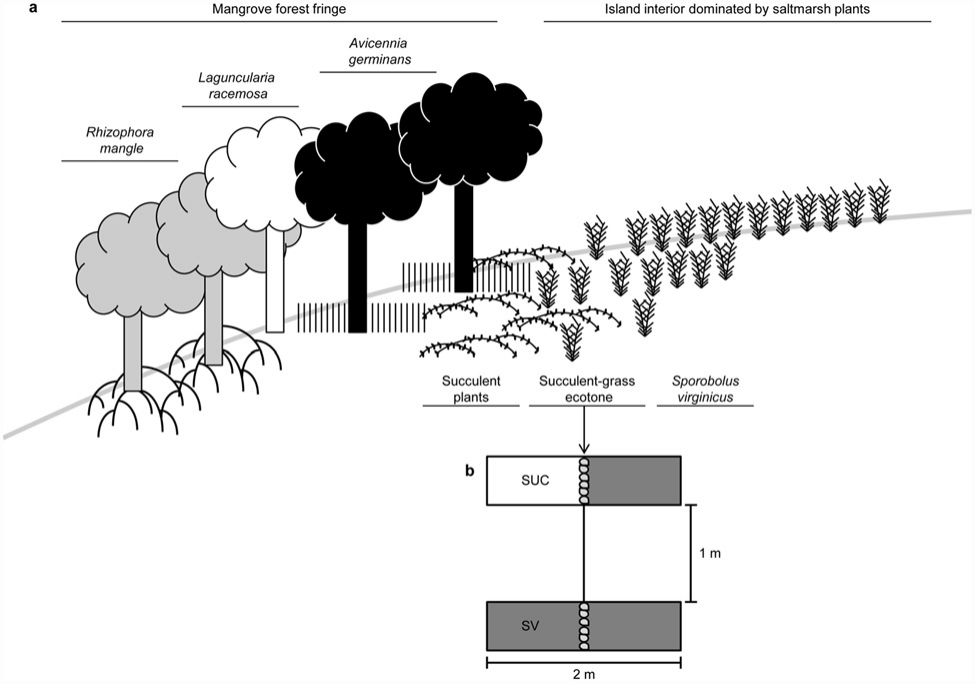
Study site diagram and experimental design. **a)** Schematic diagram (not to scale) of main plant taxa along tidal gradient at study site on Cannon Island. **b)** Design of experimental plots at succulent-grass ecotone with initial position of propagules; shading indicates saltmarsh composition: (*S*. *virginicus* = gray; succulent plants = white) for saltmarsh grass monoculture (SV) and succulent-grass ecotone (SUC) treatments.

The study was conducted on Cannon Island (25°59′33.82"N, 81°44′55.10"W), a barrier island near Naples, Florida. Mangroves fringe the tidal creek on the sheltered side of the island. Landward of the mangrove fringe, several saltmarsh taxa co-occur, providing dense groundcover and an emergent vegetational boundary (see Fig. 1a, Tables 1 and 2, and S1 and S2 Tables in Supporting Information). Near the landward edge of the mangrove forest, a conspicuous ecotone is present between succulent saltmarsh vegetation (consisting of *Sesuvium portulacastrum*, *Batis maritima*, and *Salicornia spp*.) and the saltmarsh grass, *Sporobolus virginicus*, which dominates the interior of the island (Fig. 1a). While the interior portion of Cannon Island beyond the mangrove edge is infrequently inundated by tides, water levels exceeding MHHW (mean higher high water) by at least 0.19 m can inundate the succulent-grass ecotone at the experimental site. Data from the Big Marco River (NOAA station) near our study site confirmed that high tides during the study were adequate to disperse mangrove propagules landward of the mangrove edge. Furthermore, the presence of several mangrove recruits within the saltmarsh vegetation prior to experimentation (Table 3) indicated that high tide events had previously enabled the transport of propagules into the saltmarsh landward of the mangrove fringe. However, data on the water height and velocity of ebb and flood tides at the study site are not available.

**Table 1:** Saltmarsh plant abundance within experimental plots.

Experimental Treatment	Quadrat position (m)	Saltmarsh grass	Succulent saltmarsh plants	
		*Sporobolus virginicus*	*Batis maritima*	*Sesuvium portulacastrum*
Succulent-grass	-1 to -0.5	0	36.3 (12.6)	57.5 (16.0)
	-0.5 to 0	0	31.3 (8.8)	46.3 (11.3)
	0 to 0.5	100 (0)	0	0
	0.5 to 1	100 (0)	0	0
Grass Monoculture	-1 to -0.5	72.5 (11.6)	0	0
	-0.5 to 0	100 (0)	0	0
	0 to 0.5	97.5 (2.5)	0	0
	0.5 to 1	100 (0)	0	0

Plant cover within experimental plots was documented using transects that initiated 1 m seaward and terminated 1 m landward of the 0 m position (i.e., natural conspicuous ecotone between saltmarsh grass and succulent saltmarsh plants). At each position along transects, saltmarsh plant cover was assessed within a 0.25 m^2^ quadrat, which was subdivided into 16 subsections. Experimental plots were then weeded to produce two experimental treatments: 1) succulent-grass ecotone and 2) grass monoculture; the mean percentage ground cover (±se) of saltmarsh plants within experimental plots after weeding is provided.

**Table 2:** Saltmarsh taxa frequency of occurrence.

Percentage of quadrats containing saltmarsh plants					
Quadrat position	Saltmarsh grass	Succulent saltmarsh plants			
	*Sporobolus virginicus*	*Batis maritima*	*Sesuvium portulacastrum*	*Salicornia sp*.	*Suaeda linearis*
-1 to -0.5	100	90	100	30	40
-0.5 to 0	100	90	100	10	10
0 to 0.5	100	60	100	20	0
0.5 to 1	100	70	40	10	0
1 to 1.5	100	10	50	0	0
1.5 to 2	100	10	20	0	0

Percentage of quadrats containing each saltmarsh plant prior to the manipulation of experimental plots. To assess plant abundance, transects initiating 1 m seaward and terminating 2 m landward of the 0 m position were completed for each experimental plot. At each position along transects, saltmarsh presence was assessed within a 0.25 m^2^ quadrat.

**Table 3:** Abundance of *A*. *germinans* recruits within saltmarsh adjacent to mangrove fringe.

Mean number of *A*. *germinans* m^-2^ (±se)		
Quadrat position	Seedlings	Propagules
-1 to -0.5	3.2 (0.4)	5.6 (0.7)
-0.5 to 0	6.8 (0.4)	7.6 (1.3)
0 to 0.5	0 (0)	0.8 (0.1)
0.5 to 1	0 (0)	1.6 (0.2)

Mean number of *A*. *germinans* m^-2^ (± se). To assess mangrove abundance, transects initiating 1 m seaward and terminating 1 m landward of the 0 m position were completed for each experimental plot. At each position along transects, mangrove density was assessed within a 0.25 m^2^ quadrat.

A set of field experiments was conducted at the ecotone between the two distinct saltmarsh zones (grass versus succulents) to examine mangrove propagule movement. The visual interface between the succulent saltmarsh plants and the saltmarsh grass was designated as “0 m”, and 10 experimental plots were established along this 0 m position. Plots (0.5 m wide) extended to 1 m seaward of the saltmarsh ecotone (0 m position) into the succulent-dominated zone and extended 1 m into the grass-dominated landward zone (see Fig. 1). Five experimental plots were assigned to each of two saltmarsh treatments: 1) a grass monoculture of *S*. *virginicus* extending throughout both the landward and seaward portions of the plot or 2) a succulent-grass ecotone, containing only succulent saltmarsh plants (e.g., *Batis maritima* and *Sesuvium portulacastrum*) in the seaward half of the plot and *S*. *virginicus* exclusively in the landward half of the plot (Fig. 1b). Selective weeding of plots was required to produce the treatments. Experimental plots were separated by 1 m of un-manipulated saltmarsh vegetation and treatments were interspersed to minimize the effects of any gradients in vegetation biomass or tidal inundation across the site.

After experimental plots were prepared on 27 September 2011, mature *A*. *germinans* propagules were collected by hand from trees on Cannon Island. Pericarps were carefully removed from propagules by hand. Propagules were haphazardly assigned to each of the two saltmarsh treatments: the grass monoculture (SV) or the succulent-grass ecotone (SUC). Two vibrant colors (pink, orange) of florescent spray paint differentiated *A*. *germinans* propagules from each treatment and also improved the visibility of propagules within saltmarsh vegetation after dispersal events without altering the buoyancy of propagules [25]. Using this method, the saltmarsh treatment from which propagules originated could be determined, but individual propagules could not be distinguished. Therefore, during monitoring, propagules located within plots were assumed to have originated from the plot in which they were found, and propagules that had dispersed out of experimental plots were assigned to the nearest plot of potential origin.

Propagules (24 per plot, 240 total) were placed linearly along the 0 m position within experimental plots on 28 September 2011 (Fig. 1b). Subsequently, dispersal patterns of mangrove propagules were assessed on each of two days (29 and 30 September 2011) during which spring high tides inundated the plots, providing opportunities for propagules dispersal. On each sampling date, the position of all marked propagules recovered within the search radius (~ 5 m around experimental plots) was noted, and dispersal distance of each propagule was assessed by the linear difference between its position and the 0 m location of initial propagule emplacement. We also determined the percentage of propagules that moved > 5 cm from the 0 m line in either the landward or seaward direction; this 5 cm buffer was set to minimize the effect of incidental propagule movements due to the behavior of burrowing crabs (*Uca spp*.), which were active at the site.

The search radius included areas seaward and landward of experimental plots, as well as the saltmarsh areas between and within adjacent plots. The horizontal movement of propagules (perpendicular to the intertidal gradient) was noted but could not be rigorously evaluated because propagules were not labeled within individual identifiers; therefore, the results presented here-in are for movement in the landward-seaward direction only. The search radius (~5 m perimeter around experimental plots) was constrained by time limitations; if a larger radius had been searched, the positions of some propagules carried further than the search radius may have been recorded. The authors acknowledge these limitations of the experimental design and understand that the use of this approach likely underestimated the true distances propagules moved through the saltmarsh-mangrove matrix. However, the experiment was designed to document the positions of propagules along the intertidal gradient relative to their initial position over time, and the methods employed during this field experiment achieved this objective.

After 6 weeks, on 9 November 2011, experimental *A*. *germinans* could still be differentiated from naturally occurring propagules by their pink or orange markings. At this time, the position of propagules was recorded relative to the 0 m position, as above. Furthermore, the establishment success of propagules from each experimental plot was assessed by counting the number of *A*. *germinans* from each treatment that had rooted and the number that had produced leaves.

The fate of propagules emplaced into experimental plots was evaluated by analyzing the counts of individual propagules that were 1) not recovered, 2) recovered landward, 3) recovered within 5 cm buffer, or 4) recovered seaward for each of the three sampling dates. A log-linear analysis of propagule count data was used to determine the significance of treatment, date, and the interaction between treatment and date on the fate of propagules. A separate log-linear analysis was used to determine the significance of treatment and position on the establishment success (i.e., rooting status and leaf production) of *A*. *germinans*. Additionally, the effect of saltmarsh treatment on the total number of propagules recovered vs. the total number of propagules not recovered on the last sampling date (9 November 2012) was assessed using a Chi-square test.

The position of all recovered propagules emplaced within experimental plots on each sampling date was analyzed to determine the effect of saltmarsh treatments on dispersal distance. For this statistical analysis, individual propagules were treated as subsamples, and plots were considered replicates. Data on the linear distance of propagules from their position of emplacement for all dates (29 September, 30 September, and 9 November 2011) were analyzed using repeated measures analysis of variance (RMANOVA); these data satisfied the assumptions of RMANOVA according to visual inspection of residual plots, and the results of Kolmogorov—Smirnov (normality), Levene (homogeneity of variance), and Mauchley (sphericity) tests. When a significant treatment effect (*p* < 0.05) was detected, a Fisher LSD post-hoc test was performed. Patterns of propagule dispersal and establishment were analyzed using Statistica 10.

## Results

High tide events capable of dispersing propagules repeatedly occurred at the study site following propagule emplacement into experimental plots. A total of 45 high tide events were predicted to have exceeded MHHW at our study site throughout the study duration, 29 September through 9 November 2011 [41]. Tidal inundation of experimental plots and dispersal of propagules away from their initial position were visually evident after 1 d. Even so, recovery of marked propagules emplaced into saltmarsh boundary treatments was remarkably high. Across all plots, 97.1% and 96.7% of propagules were recovered 1 and 2 d after emplacement, respectively (Table 4). Six weeks after initial emplacement into plots, a total of 192 propagules were recovered; at this time, 85% of propagules originally emplaced into grass monoculture plots were recovered compared to 75% of those emplaced into succulent-grass ecotone plots (Table 4). The number of propagules recovered from the two saltmarsh treatments (succulent-grass vs. grass monoculture) in November was significantly different (χ^2^ = 3.75, df = 1, *p* = 0.05), suggesting that a greater percentage of propagules dispersed beyond the searched area when emplaced into succulent-grass versus grass monoculture plots along the mangrove—saltmarsh ecotone.

**Table 4:** Fate of *A*. *germinans* propagules in experiment.

Date	Treatment	Not recovered	Seaward	Within Buffer	Landward
29 September	SV	2.5	30.0	45.8	21.7
29 September	SUC	3.3	56.7	28.3	11.7
30 September	SV	3.3	28.3	42.5	25.8
30 September	SUC	3.3	49.2	24.2	23.3
9 November	SV	15.0	23.3	33.3	28.3
9 November	SUC	25.0	37.5	15.0	22.5

The percentages of propagules from fall 2011 experiments with each of the four possible fates by date are presented for experimental plots assigned to two saltmarsh treatments (n = 5 plots per treatment): saltmarsh grass monoculture (SV) and succulent-grass ecotone (SUC) treatments. See text for complete description of fate categories and treatments.

**Fig 2:**
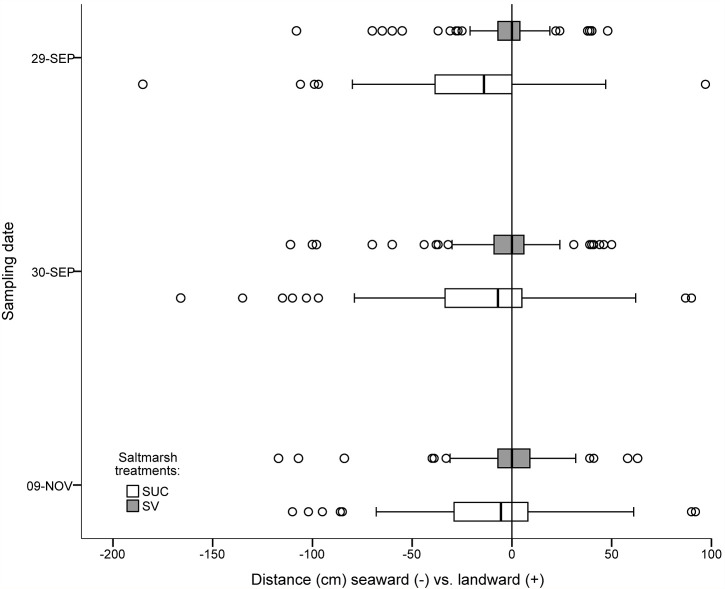
Spatial dispersion of *A*. *germinans* relative to initial position. Box-plot with data indicating the distance from initial position (0 m) at which marked *A*. *germinans* were recovered for saltmarsh grass monoculture (SV = gray) and succulent-grass ecotone (SUC = white) treatments. Data include all *A*. *germinans* regardless of rooting status. For each box-plot, median distance (bolded line) and outliers (circles) are shown.

**Fig 3:**
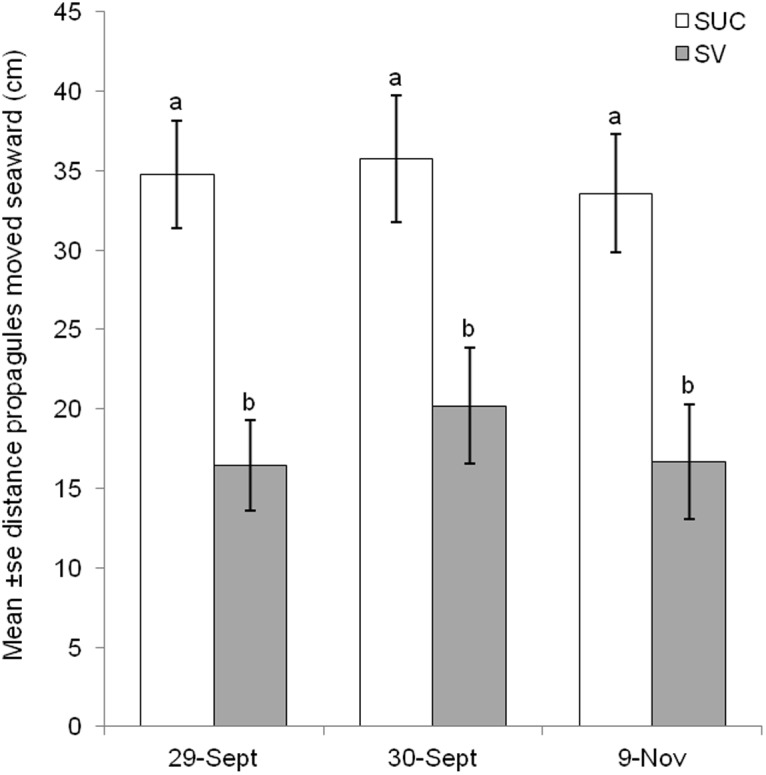
Distance moved by *A*. *germinans* propagules that dispersed seaward. Mean distance ± se (cm) seaward of initial position at which *A*. *germinans* propagules were recovered for saltmarsh grass monoculture (SV) or succulent-grass ecotone (SUC) treatments on three dates. Different letters above bars represent significantly different distances (RMANOVA).

Propagules from each of the 10 experimental plots in the two saltmarsh treatments dispersed both seaward and landward of their starting position (Fig. 2). But, across all plots, propagules most frequently dispersed in the seaward direction. Furthermore, the mean distance moved seaward was significantly greater for propagules initially emplaced into succulent-grass plots compared to those from grass monoculture plots (RMANOVA, F_1,8_ = 11.118, *p* = 0.01, Fig. 3). On the first day after emplacement, propagules originating from the succulent-grass plots and which subsequently moved seaward were recovered 34.8 (±3.4) cm [mean (± se)] away from the starting position. In contrast, propagules moving seaward and originating from grass monoculture plots were recovered 16.5 (±2.8) [mean (± se)] cm from their initial position.

The fate of propagules, as evaluated by counts of individual propagules that were 1) not recovered, 2) recovered landward, 3) recovered within a 5 cm buffer, or 4) recovered seaward, on each of the three sampling dates, clearly indicated that saltmarsh boundary type influenced the number of propagules that were dispersed in the seaward direction. Across all dates, ~63% of propagules that were transported beyond a 5 cm buffer (see Methods) moved seaward while ~37% were recorded landward of the buffer (Table 1). After only one day, a greater proportion of propagules originating within succulent-grass plots was transported seaward than that from grass monoculture plots. The best model for predicting the fate of propagules included the interaction between fate and saltmarsh boundary treatment and the interaction between fate and sampling date (Max likelihood χ^2^ = 3.091, df = 8, *p* = 0.93). The inclusion of the interaction between date and treatment did not significantly improve the model (χ^2^ = 0.172, df = 2, *p* = 0.92). The fate of propagules (Table 4) was influenced significantly by saltmarsh treatment (χ^2^ = 44.66, df = 3, *p* < 0.0001). Also, a significant effect of date on fate of propagules was also detected (χ^2^ = 63.96, df = 6, *p* < 0.0001) resulting from the reshuffling of some propagules by numerous tidal events over the study duration and the increased percentage of propagules that exited the experimental site after 6 weeks (Table 4). Both lines of evidence: the percentage of propagules transported seaward (Table 4) and distance propagules moved seaward (Fig. 3), indicate that seaward transport of propagules was significantly reduced when saltmarsh grass alone was present.

**Fig 4:**
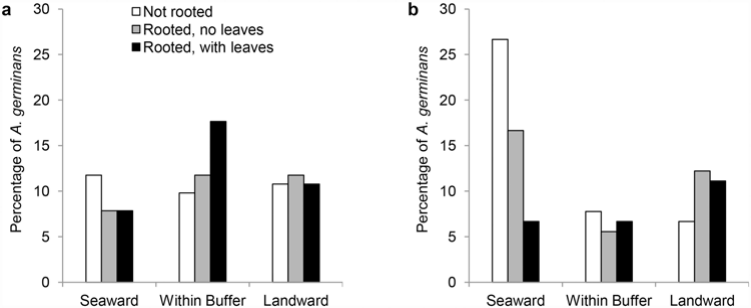
Establishment of *A*. *germinans* on 9 November 2012. Initial establishment success was evaluated by categorizing the rooting status and leaf development of *A*. *germinans* six weeks after propagule emplacement into two saltmarsh treatment a) saltmarsh grass monoculture (SV) and b) succulent-grass ecotone (SUC). The percentages of *A*. *germinans* propagules in the three categories of seedlings establishment at three positions relative to the starting position (0 m) within the saltmarsh are presented for each of the saltmarsh treatments.

The composition of the experimental saltmarsh boundaries into which propagules were emplaced also influenced spatial patterns of mangrove establishment. By 6 weeks, 50.8% of all propagules had successfully rooted (Fig. 4). Of the total number of propagules initially emplaced into all plots, 17.1% rooted within the 5 cm buffer, and 18.3% and 15.4% rooted in a position landward and seaward, respectively, of their starting location (Fig. 4). A log-linear analysis revealed that the best model (Max likelihood χ^2^ = 4.381, df = 6, *p* = 0.62) for predicting the establishment status (i.e., not rooted, rooted without leaves, and rooted with leaves) of *A*. *germinans* on 9 November included the interaction (χ^2^ = 9.320, df = 4, *p* = 0.05) between *A*. *germinans* establishment status and position (within buffer, landward, or seaward). The best fit model also included the interaction (χ^2^ = 10.082, df = 2, *p* = 0.01) between *A*. *germinans* position and the composition of the saltmarsh boundary (i.e., succulent-grass or grass monoculture) within which *A*. *germinans* propagules were originally emplaced before being transported. Over a 6 week period, although a smaller percentage of *A*. *germinans* propagules moved landward versus seaward, a substantially higher percentage (~72%) of propagules that were recovered landward successfully rooted compared to that (~51%) for those recovered from seaward positions (Fig. 4). Additionally, the mean position of *A*. *germinans* propagules emplaced within grass monoculture plots which then rooted was slightly landward of their starting position [mean (± se) = 0.4 (± 2.4) cm] within the buffer. Conversely, propagules from succulent-grass plots rooted a mean (± se) distance of 6.7 (± 5.1) cm seaward of the starting position. On the last sampling date, we also observed a trend towards greater leaf production by *A*. *germinans* seedlings (71% produced leaves) from the grass monoculture treatment compared to leaf production by seedlings (52%) that were emplaced into the succulent-grass ecotone treatment. Finally, *A*. *germinans* propagules that had been emplaced into grass monoculture plots produced a slightly higher number of leaves than that recorded for individuals emplaced into succulent-grass plots [mean (± se), 1.4 (± 0.1) and 1.0 (± 0.2) leaves per seedling, respectively].

## Discussion

Factors influencing an inland niche shift of the black mangrove emerge from our quantification of landward movement of mangrove propagules upon encountering saltmarsh boundaries. Our study demonstrates that biotic boundaries erected by saltmarsh plants play a key role in directing the initial sequence of landward migration of mangroves expected under conditions of increasing sea-level. More specifically, hydrochorous transport vectors (e.g., high tides or storm events) and structural features of saltmarsh plants combine to determine permeability of inland boundaries to dispersing mangrove propagules. By recording both propagule retention and seedling establishment at landward mangrove margins we newly identified processes that contribute to successful mangrove persistence during inland migration. Our work provides empirical assessment of responses by early life-history stages of the black mangrove to the spatial arrangement of co-occurring coastal taxa thereby adding insight into the adaptive capacity for mangroves to respond to challenges from climate change.

In agreement with *in situ* work on mangrove dispersal [25], mangrove propagules in our experiments generally dispersed in a seaward direction, opposite to the landward shift in distribution required to maintain optimal intertidal position as sea-level rises [19, 20]. The dominance of seaward dispersal of mangrove propagules during our study remains a striking feature of tidal influences on mangrove recruitment. This experiment was initiated during the wet season, and sheet-flow runoff of rainwater during the experiment may have contributed to the seaward dispersal of propagules [25]. This finding implies that responses (i.e., cross boundary transport) by black mangroves to rising sea-level may be accomplished by only a small proportion of the potential cohort of recruits. Together the new information suggests an overall scenario of mangrove inland migration being linked to the fate of the subset of propagules that moves directionally opposite to most during high tides and successfully disperses landward across biotic boundaries.

Although propagules emplaced into either boundary condition, i.e., grass monoculture or succulent-grass, generally moved seaward, the influence of saltmarsh taxa on the inland movement of the remaining propagules varied in a consistent way. Notably, propagule retention was improved at the saltmarsh boundary composed of monocultures of the saltmarsh grass, *Sporobolus virginicus*, because both frequency and distance of seaward dispersal of propagules was reduced compared to the boundary containing succulent plants. Where succulents were present, comparatively higher proportions of propagules transported seaward led to a reduced abundance of potential colonizers remaining at the leading edge of mangrove migration. Propagule dispersal through saltmarsh plants (i.e., saltmarsh permeability) was likely influenced by the physical characteristics of the saltmarsh taxa, such as structural complexity [34], height (S1 Table) and biomass (S2 Table). These results imply that, under similar tidal conditions, the probability of propagules moving inland should be mechanistically linked to patch (taxa) composition of saltmarsh ecotones.

Various types of saltmarsh-mangrove boundaries occur throughout the Caribbean and Gulf of Mexico [20, 25, 28, 30]. Therefore, the permeability of saltmarsh boundaries by mangrove propagules at other coastal locations may differ from the patterns observed at our study site on Cannon Island. Boundary permeability likely depends upon site-specific factors, such as the spatial configuration (e.g., zonation) of saltmarsh-mangrove boundaries. Moreover, propagule dispersal patterns are likely a function of the relative velocity of ebb and flood tides; asymmetrical ebb and flood tidal forces could influence both the distance and direction of propagule dispersal. Additionally, permeability of saltmarsh boundaries is likely to differ depending upon the mangrove taxa present at the site due to the effect of morphological characteristics (e.g., shape and size) of mangrove propagules on dispersal [25, 26, 31]. For example, *Rhizophora mangle* propagules, which are considerably larger and distinctly different in shape than *A*. *germinans* [31], may become entrapped within succulent saltmarsh plants [42]; whereas, we documented dispersal of *A*. *germinans* propagules through succulent saltmarsh vegetation at our study site.

Landward transport of mangrove propagules at the saltmarsh boundary occurred within days; yet a pattern demonstrating inland recruitment persisted for at least 6 weeks, after which time most propagules became established seedlings. Moreover, a proportional increase in seedling establishment and leaf production was recorded for propagules that dispersed landward relative to seaward. These observations are aligned with Rabinowitz [31] who documented enhanced rooting of mangrove propagules during stranding. Furthermore, these observations and the presence of fully reproductive black mangroves interspersed within upland vegetation at even higher tidal elevations than that at our study site (J.M. Peterson, *personal observations*), suggest that some propagules transported landward are able to survive and ultimately achieve reproductive success. As appears to be true for mangroves, studies of migration by terrestrial plants challenged by climate drivers have also reported examples of directional dispersal running counter to the predicted direction of a niche shift [2, 15, 16]. For example, uphill migration of montane plants [2, 14, 43] has received considerable attention; seeds must disperse against gravitational forces and successfully establish up-slope for populations to keep pace with suitable climate conditions [44]. Directional dispersal of seeds uphill is assisted by herbivores [45]. Mack [43] noted that seeds of the montane plant, *Aglaia* aff. *flavida* were preferentially deposited uphill by *Casuarius bennetti* while, in contrast, most undispersed seeds moved downhill. Thus spatial shifts of both this montane plant and black mangrove appear to depend upon a comparatively small subset of propagules that are transported to, and successfully establish in, suitable sites.

Previously unavailable information on the direction of mangrove propagule dispersal across saltmarsh boundaries and spatial patterns of seedling establishment in the context of landscape features emerged from our experiments. Building on these findings, conditions under which boundary permeability to tidally-dispersed mangrove propagules and subsequent recruitment patterns might be altered can be identified. For instance, the potential for mangroves to be transported landward and establish at comparatively higher tidal elevations should vary if dispersal vectors or saltmarsh community composition are modified because of associated impacts to boundary function [35, 36, 39]. Our results suggest that adequate records of tidal inundation levels and detailed spatial representations of coastal landscapes will both be required to advance models of forecasted mangrove distributional shifts. We have demonstrated the importance of including the taxonomic composition of saltmarsh taxa in such spatial representations of coastal landscapes, but anthropogenic structures along shorelines may also obstruct or facilitate propagule dispersal and warrant inclusion in predictive models [46–47].

Climate change is expected to increase the frequency/intensity of storms [48], which may have far-reaching hydrological impacts [49] and accordingly modify dispersal trajectories of mangrove propagules. A key factor determining the relative importance of biotic boundaries in modulating the niche shift of black mangroves will likely be the frequency and amplitude of high water tidal events during time of propagule production. In some cases, tidal surges could transport mangrove propagules over saltmarsh canopies into areas further inland than locations where propagules would be deposited by typical tides. Under such circumstances, saltmarsh boundary permeability may have reduced influence on landward dispersal of mangrove propagules.

Additionally, propagule dispersal may be altered if the ecotone between saltmarsh succulents and grasses itself responds dynamically to climate drivers by shifting landward [50]. As structural features of boundaries change, so too should the spatial patterns of mangrove propagule dispersal and seedling establishment. Unfortunately, a relatively poor understanding of coordinated responses underlying the landward progression of mangrove and saltmarsh distributions hinders more detailed predictions of the tempo of what will likely involve distributional shifts of multiple taxa.

Furthermore, complex biotic interactions between mangroves and saltmarsh taxa may operate not only during mangrove dispersal and recruitment, as shown here, but also during later life stages [28]. In this study, a relatively small proportion (20%) of propagules emplaced into experimental plots was not recovered and may have been consumed by herbivores (alternatively, those propagules may have dispersed beyond the search radius). While not the focus of this study, ecological interactions between saltmarsh and mangrove recruits, such as associational resistance/susceptibility to herbivory, have been demonstrated to operate in a variety of plant communities and could potentially influence mangrove expansion into saltmarsh-dominated areas. The study of mangrove-saltmarsh interactions is complicated by the fact that ecological filters such as abiotic constraints (i.e., water stress and flooding frequency) on mangrove growth and survival are dictated by elevational and latitudinal gradients, which can influence whether biotic interactions between mangrove seedlings and saltmarsh plants are competitive or facilitative [28]. Ecological interactions between mangrove and saltmarsh taxa are multifaceted and warrant additional study given their potential implications for distributional shifts of coastal plant taxa in response to climate change.

In summary, our results suggest that interplay between tidal transport and physical attributes of saltmarsh vegetation assumes a critical role in directing the initial phase of shifting mangrove distributions and thus the capacity of black mangroves to respond to rising sea-level. These combined findings imply that any modification of tidal conditions or changes in plant composition of intertidal/supratidal landscapes through which propagules must disperse should impact the rate of successful mangrove migration as a consequence of altered mangrove dispersal and boundary permeability. The next challenge is to evaluate the capacity of mangroves to migrate inland as the coastal landscape is rearranged not only by rising sea-level but also other climate drivers.

## Supporting Information

S1 Table Mean ±se height (cm) of saltmarsh plants on Cannon Island in August 2009. (PDF)

S2 Table Mean (±se) biomass of saltmarsh plants (g dry weight x m^-2^) by distance (m) from mangrove tree line at two locations (north and south) on Cannon Island in November 2009. (PDF)

## References

[1] Parmesan C, Yohe G. A globally coherent fingerprint of climate change impacts across natural systems. Nature 2003;421: 37–42. 12511946 10.1038/nature01286

[2] Walther G-R, Beißner S, Burga CA. Trends in the upward shift of alpine plants. J Veg Sci 2005;16: 541–548.

[3] Pearson RG, Dawson TP. Predicting the impacts of climate change on the distribution of species: are bioclimate envelope models useful? Glob Ecol Biogeogr 2003;12: 361–371.

[4] Keith SA, Herbert RJH, Norton PA, Hawkins SJ, Newton AC. Individualistic species limitations of climate-induced range expansions generated by meso-scale dispersal barriers. Divers Distrib 2011;17: 275–286.

[5] Zhu K, Woodall CW, Clark JS. Failure to migrate: lack of tree range expansion in response to climate change. Glob Change Biol 2012;18: 1042–1052.

[6] Bellard C, Bertelsmeier C, Leadley P, Thuiller W, Courchamp F. Impacts of climate change on the future of biodiversity. Ecol Lett 2012;15: 365–377. 22257223 10.1111/j.1461-0248.2011.01736.x PMC3880584

[7] Thomas CD, Cameron A, Green RE, Bakkenes M, Beaumont LJ, Collingham YC, et al Extinction risk from climate change. Nature 2004;427: 145–148. 14712274 10.1038/nature02121

[8] Renton M, Childs S, Standish R, Shackelford N. Plant migration and persistence under climate change in fragmented landscapes: Does it depend on the key point of vulnerability within the lifecycle? Ecol Model 2013;249: 50–58.

[9] Berger U, Rivera-Monroy VH, Doyle TW, Dahdouh-Guebas F, Duke NC, Fontalvo-Herazo ML, et al Advances and limitations of individual-based models to analyze and predict dynamics of mangrove forests: A review. Aquat Bot 2008;89: 260–274.

[10] Teh SY, DeAngelis DL, Sternberg LDL, Miralles-Wilhelm FR, Smith TJ, Koh HL. A simulation model for projecting changes in salinity concentrations and species dominance in the coastal margin habitats of the Everglades. Ecol Model 2008;213: 245–256.

[11] Jiang J, DeAngelis D, Anderson G, Smith T III. Analysis and simulation of propagule dispersal and salinity intrusion from storm surge on the movement of a marsh-mangrove ecotone in South Florida. Estuaries Coasts 2014;37: 24–35.

[12] de Lange WP, de Lange PJ. An appraisal of factors controlling the latitudinal distribution of mangrove (*Avicennia marina* var. *resinifera*) in New Zealand. J Coastal Res 1994;10: 539–548.

[13] Osland MJ, Enwright N, Day RH, Doyle TW. Winter climate change and coastal wetland foundation species: salt marshes vs. mangrove forests in the southeastern United States. Glob Change Biol 2013;19: 1482–1494. 10.1111/gcb.12126 23504931

[14] Beckage B, Osborne B, Gavin DG, Pucko C, Siccama T, Perkins T. A rapid upward shift of a forest ecotone during 40 years of warming in the Green Mountains of Vermont. Proc Natl Acad Sci U S A 2008;105: 4197–4202. 10.1073/pnas.0708921105 18334647 PMC2393766

[15] Breshears DD, Huxman TE, Adams HD, Zou CB, Davison JE. Vegetation synchronously leans upslope as climate warms. Proc Natl Acad Sci U S A 2008;105: 11591–11592. 10.1073/pnas.0806579105 18697950 PMC2575300

[16] Harsch MA, Hulme PE, McGlone MS, Duncan RP. Are treelines advancing? A global meta-analysis of treeline response to climate warming. Ecol Lett 2009;12: 1040–1049. 10.1111/j.1461-0248.2009.01355.x 19682007

[17] Cavanaugh KC, Kellner JR, Forde AJ, Gruner DS, Parker JD, Rodriguez W, et al Poleward expansion of mangroves is a threshold response to decreased frequency of extreme cold events. Proc Natl Acad Sci U S A 2013;111: 723–727. 10.1073/pnas.1315800111 24379379 PMC3896164

[18] Saintilan N, Wilson NC, Rogers K, Rajkaran A, Krauss KW. Mangrove expansion and salt marsh decline at mangrove poleward limits. Glob Change Biol 2014;20: 147–157. 10.1111/gcb.12341 23907934

[19] Adam P. Saltmarshes in a time of change. Environ Conserv 2002;29: 39–61.

[20] Krauss K, From A, Doyle T, Doyle T, Barry M. Sea-level rise and landscape change influence mangrove encroachment onto marsh in the Ten Thousand Islands region of Florida, USA. J Coast Conser 2011;15: 629–638.

[21] Gaiser EE, Zafiris A, Ruiz PL, Tobias FAC, Ross MS. Tracking rates of ecotone migration due to salt-water encroachment using fossil mollusks in coastal South Florida. Hydrobiologia 2006;569: 237–257.

[22] Ellison JC. Long-term retrospection on mangrove development using sediment cores and pollen analysis: A review. Aquat Bot 2008;89: 93–104.

[23] Cohen MCL, Lara RJ. Temporal changes of mangrove vegetation boundaries in Amazonia: Application of GIS and remote sensing techniques. Wetl Ecol Manag 2003;11: 223–231.

[24] Eslami-Andargoli L, Dale P, Sipe N, Chaseling J. Mangrove expansion and rainfall patterns in Moreton Bay, Southeast Queensland, Australia. Estuar Coast Shelf Sci 2009;85: 292–298.

[25] Sousa WP, Kennedy PG, Mitchell BJ, Ordonez BM. Supply-side ecology in mangroves: Do propagule dispersal and seedling establishment explain forest structure? Ecol Monogr 2007;77: 53–76.

[26] De Ryck DJR, Robert EMR, Schmitz N, Van der Stocken T, Di Nitto D, Dahdouh-Guebas F, et al Size does matter, but not only size: Two alternative dispersal strategies for viviparous mangrove propagules. Aquat Bot 2012;103: 66–73.

[27] Gilman E, Ellison J, Coleman R. Assessment of mangrove response to projected relative sea-level rise and recent historical reconstruction of shoreline position. Environ Monit Assess 2007;124: 105–130. 17171293 10.1007/s10661-006-9212-y

[28] Guo H, Zhang Y, Lan Z, Pennings SC. Biotic interactions mediate the expansion of black mangrove (*Avicennia germinans*) into salt marshes under climate change. Glob Change Biol 2013;19: 2765–2774. 10.1111/gcb.12221 23580161

[29] Breitfuss MJ, Connolly RM, Dale PER. Mangrove distribution and mosquito control: transport of *Avicennia marina* propagules by mosquito—control runnels in southeast Queensland saltmarshes. Estuar Coast Shelf Sci 2003;56: 573–579.

[30] Alleman L, Hester M. Reproductive ecology of black mangrove (*Avicennia germinans*) along the Louisiana coast: Propagule production cycles, dispersal limitations, and establishment elevations. Estuaries Coasts 2011;34: 1068–1077.

[31] Rabinowitz D. Dispersal properties of mangrove propagules. Biotropica 1978:10: 47–57.

[32] Clarke PJ. Dispersal of grey mangrove (*Avicennia marina*) propagules in southeastern Australia. Aquat Bot 1993;45: 195–204.

[33] López-Medellín X, Ezcurra E, González-Abraham C, Hak J, Santiago LS, Sickman JO. Oceanographic anomalies and sea-level rise drive mangroves inland in the Pacific coast of Mexico. J Veg Sci 2011;22: 143–151.

[34] Peterson JM, Bell SS. Tidal events and salt-marsh structure influence black mangrove (*Avicennia germinans*) recruitment across an ecotone. Ecology 2012;93: 1648–1658. 22919911 10.1890/11-1430.1

[35] Cadenasso ML, Pickett STA, Weathers KC, Bell SS, Benning TL, Carreiro MM, et al An interdisciplinary and synthetic approach to ecological boundaries. BioScience 2003;53: 717–722.

[36] Cadenasso ML, Pickett STA, Weathers KC, Jones CG. A framework for a theory of ecological boundaries. BioScience 2003;53: 750–758.

[37] Pépino M, Rodríguez MA, Magnan P. Fish dispersal in fragmented landscapes: a modeling framework for quantifying the permeability of structural barriers. Ecol Appl 2012;22: 1435–1445. 22908704 10.1890/11-1866.1

[38] Schultz CB, Crone EE. Edge-mediated dispersal behavior in a prairie butterfly. Ecology 2001;82: 1879–1892.

[39] Cadenasso ML, Pickett STA. Effect of edge structure on the flux of species into forest interiors. Conserv Biol 2001;15: 91–97.

[40] Castro J, Zamora R, Hódar JA. Mechanisms blocking *Pinus sylvestris* colonization of Mediterranean mountain meadows. J Veg Sci 2002;13(5):725–731.

[41] NOAA NWS, Southern Regional Headquarters (2013).

[42] Donnelly M, Walters L. Trapping of *Rhizophora mangle* propagules by coexisting early successional species. Estuaries Coasts 2014;37: 1562–1571

[43] Mack AL. Distance and non-randomness of seed dispersal by the dwarf cassowary *Casuarius bennetti* . Ecography 1995;18: 286–295.

[44] Walther G-R, Post E, Convey P, Menzel A, Parmesan C, Beebee TJC, et al Ecological responses to recent climate change. Nature 2002;416: 389–395. 11919621 10.1038/416389a

[45] Grinnell J. Up-Hill Planters. The Condor 1936;38: 80–82.

[46] Gilman E, Ellison J, Coleman R. Assessment of mangrove response to projected relative sea-level rise and recent historical reconstruction of shoreline position. Environ Monit Assess 2007;124: 105–130 17171293 10.1007/s10661-006-9212-y

[47] Breitfuss MJ, Connolly RM, Dale PER. Mangrove distribution and mosquito control: transport of *Avicennia marina* propagules by mosquito—control runnels in southeast Queensland saltmarshes. Estuar Coast Shelf Sci 2003;56: 573–579

[48] Knutson TR, McBride JL, Chan J, Emanuel K, Holland G, Landsea C, et al Tropical cyclones and climate change. Nature Geosci 2010;3: 157–163.

[49] Harley CDG, Randall Hughes A, Hultgren KM, Miner BG, Sorte CJB, Thornber CS, et al The impacts of climate change in coastal marine systems. Ecol Lett 2006;9: 228–241. 16958887 10.1111/j.1461-0248.2005.00871.x

[50] Wasson K, Woolfolk A, Fresquez C. Ecotones as indicators of changing environmental conditions: Rapid migration of salt marsh-upland boundaries. Estuaries Coasts 2013;36: 654–664.

