# Supplementary material for: Saltmarsh Boundary Modulates Dispersal of Mangrove Propagules: Implications for Mangrove Migration with Sea-Level Rise
Source: PLoS One. 2015 Mar 11;10(3):e0119128. doi: 10.1371/journal.pone.0119128 (PMC4356570; doi:10.1371/journal.pone.0119128)
Supplement: S1 Table — (PDF) [file pone.0119128.s001.pdf]

In order to characterize saltmarsh vegetation on Cannon Island, the height (cm) of saltmarsh plants was surveyed at two sites (north and south) in August 2009 (Table S1).

Table S1: Mean ±se height (cm) of saltmarsh plants on Cannon Island in August 2009

Site	Saltmarsh plants	Mean (±se) height
North	*Sporobolus virginicus*	31.9 (1.5)
	Sesuvium portulacastrum	11.3 (1.1)
	Batis maritima	26.2 (5.7)
South	*Sporobolus virginicus*	33.1 (1.5)
	Sesuvium portulacastrum	17.7 (2.0)
	Batis maritima	24.9 (9.1)
