# Supplementary material for: Saltmarsh Boundary Modulates Dispersal of Mangrove Propagules: Implications for Mangrove Migration with Sea-Level Rise
Source: PLoS One. 2015 Mar 11;10(3):e0119128. doi: 10.1371/journal.pone.0119128 (PMC4356570; doi:10.1371/journal.pone.0119128)
Supplement: S2 Table — (PDF) [file pone.0119128.s002.pdf]

The above-ground biomass of saltmarsh plants was surveyed at two sites (north and south) on Cannon Island in November 2009; samples (n=96) were collected within a 0.10 m x 0.10m quadrat. Above-ground biomass was clipped to the sediment level, harvested, and frozen until processed. Saltmarsh biomass samples were rinsed and dried (60°C) to constant weight (g). Mean above-ground biomass (g dry weight m^{-2}) was calculated for each plant at each sampling position (n=6) along transects (Table S2).

Table S2: Mean (±se) biomass of saltmarsh plants (g dry weight m^{-2}) by distance (m) from mangrove tree line at two locations (north and south) on Cannon Island in November 2009.

**NORTH**	**0m**	**5m**	**10m**	**15m**	**20m**	**25m**
Sporobolus virginicus	178 (39)	168 (31)	209 (34)	381 (89)	121 (43)	2 (0)
Sesuvium portulacastrum	85 (28)	133 (54)	121 (56)	0	0	0
Batis maritima	214 (63)	104 (98)	311 (0)	0	0	0
Paspalum sp.	0	325 (314)	1664 (0)	147 (117)	721 (0)	219 (0)
Spartina patens	0	1 (0)	77 (46)	90 (81)	46 (17)	3426 (0)
Suaeda linearis	0	0	0	0	138 (0)	0
Sporobolus domingensis	0	0	0	0	0	0
Borrichia frutescens	0	0	0	0	0	267 (0)
Solidago sempervirens	0	0	0	0	0	29 (0)
**SOUTH**	**0m**	**5m**	**10m**	**15m**	**20m**	**25m**
Sporobolus virginicus	178 (33)	319 (69)	449 (195)	281 (168)	266 (218)	164 (144)
Sesuvium portulacastrum	179 (32)	90 (27)	141 (0)	0	16 (0)	311 (76)
Batis maritima	39 (15)	0	0	0	0	0
Paspalum sp.	0	663 (0)	1267 (219)	1662 (551)	522 (410)	0
Spartina patens	0	0	88 (47)	492 (362)	580 (354)	517 (343)
Suaeda linearis	0	0	74 (0)	0	0	0
Sporobolus domingensis	0	0	0	0	0	2626 (0)
Borrichia frutescens	0	0	0	0	0	0
Solidago sempervirens	0	0	0	0	0	0
